# Visuomanual Vertical Prism Adaptation: Aftereffects on Visuospatial and Auditory Frequency Representations

**DOI:** 10.3389/fpsyg.2022.850495

**Published:** 2022-04-26

**Authors:** Clémence Bonnet, Bénédicte Poulin-Charronnat, Vincent Ardonceau, Cyril Sirandré, Patrick Bard, Carine Michel

**Affiliations:** ^1^INSERM UMR 1093-CAPS, Université Bourgogne Franche-Comté, UFR des Sciences du Sport, Dijon, France; ^2^LEAD, CNRS UMR 5022, Université Bourgogne Franche-Comté, Dijon, France

**Keywords:** vertical prism adaptation, auditory frequency representation, vertical visual representation, sensorimotor plasticity, crossmodal aftereffects

## Abstract

Sensorimotor aftereffects have been widely studied after lateral prism adaptation but not after vertical prism adaptation. It is thus well-known that lateral prism adaptation produces aftereffects on visuospatial representation and, recently, on auditory perception. This study aimed to explore the sensorimotor after-effects of vertical prism adaptation as well as its aftereffects on vertical visuospatial representation (Experiment 1) and on auditory frequency representation (Experiment 2). The experimental procedure was similar in both experiments: before and after prism adaptation to an upward or a downward optical deviation, healthy young participants performed an visual open-loop pointing task and a visual (Experiment 1) or an auditory (Experiment 2) perceptual bisection task. In the visual task, the participants had to indicate if they perceived the bisection as higher or lower than the true center of a line. In the auditory task, the participants had to indicate if they perceived the target auditory frequency closer to the low or the high limit of an auditory interval. For sensorimotor aftereffects, pointing errors were computed by means of a vertical touchscreen. For the perceptual bisection task, we measured the percentage of “down” (Experiment 1) or “low” responses (Experiment 2), and we computed the visual (Experiment 1) or the auditory (Experiment 2) subjective center for each participant. Statistical analyses were carried out separately for each optical deviation in each experiment. Sensorimotor aftereffects were observed in both experiments, in the opposite direction to the optical deviation (all *p*s < 0.01). No significant aftereffects occurred on visuospatial representation (all *p*s > 0.5), whereas the percentage of “low” responses and the auditory subjective center significantly increased after adaptation to a downward optical deviation (all *p*s < 0.05). Unlike lateral prism adaptation aftereffects that have been previously shown in both visuospatial horizontal representation and auditory frequency representation, aftereffects of vertical prism adaptation occurred in the auditory frequency representation but not in the vertical visuospatial representation. These results suggest that both vertical and lateral prism adaptations share a common substrate dedicated to the auditory modality (probably the temporal cortex), and that vertical adaptation does not act on the neural substrate of vertical visuospatial representation.

## Introduction

Since the end of the 19th century, researchers have been using lateral prism adaptation, which consists of pointing to visual targets while wearing prisms that shift the visual field laterally (i.e., leftward or rightward) in order to study sensorimotor adaptation (for a review, see Kornheiser, 1976). This experimental paradigm consists of three successive steps, namely measurement of baseline performance (i.e., pretest), exposure to prisms, and measurement of aftereffects (i.e., posttest). Before prism exposure, participants correctly reach the visual target. At the beginning of exposure, participants make pointing errors in the direction of the optical deviation. As the prismatic exposure progresses, participants gradually correct their errors until they perform an accurate behavior. At prism removal, pointing errors are shifted in the opposite direction of the optical deviation (e.g., Redding et al., 2005). These sensorimotor aftereffects, which testify of sensorimotor adaptation, can be explained by visual, proprioceptive, and motor control changes (for a review, see Kornheiser, 1976).

Beyond the sensorimotor level, aftereffects of prism adaptation extend to cognition (for review, see Michel, 2006, 2016). The term “cognition” refers to a large panel of our mental abilities, independent of the visuomanual coordination directly involved in the development of prism adaptation. For instance, cognitive aftereffects are key in attention, judgment, or spatial representation. The latter, which corresponds to a mental image of the space mapped across the brain, is classically assessed by the line-bisection task. In its manual version, participants place a mark at the center of a horizontal line; in its perceptual version (i.e., Landmark task), participants have to judge whether a horizontal line is transected to the left or to the right of its true center. Neglect patients, who present an inability to signal, respond, or orient toward contralesional stimuli after right hemispheric stroke (e.g., Heilman et al., 2000), typically bisect horizontal lines to the right of the veridical center. They show a mental underrepresentation of the left part of space and a mental overrepresentation of the right part of space (e.g., Milner and Harvey, 1995). These results correspond to those obtained in a line-extension task, in which neglect patients show a leftward overextension when they have to double horizontal line toward the left. Altogether, these studies are in accordance with Bisiach’s theory of anisometry of space representation: neglect patients present a behavioral overrepresentation of the left part of space that reflects a mental underrepresentation of this same part (Bisiach et al., 1994, 1998b). Healthy participants tend to bisect horizontal lines to the left of the veridical center; this phenomenon is called “pseudoneglect,” in reference to the behavior of neglect patients (Bowers and Heilman, 1980; McCourt and Jewell, 1999). In other terms, healthy people exhibit a mental overrepresentation of the left part of space and a mental underrepresentation of the right part of space, which result in a behavioral underrepresentation of the left part of space and a behavioral overrepresentation of the right part of space, according to Bisiach’s theory of anisometry (Bisiach et al., 1998b). Pseudoneglect is due to the dominance of the right hemisphere in visuospatial processes (e.g., Zago et al., 2017), and its magnitude varies not only between individuals but also according to several factors such as age, presence of lateralized cues, spatial location, and length of the line (Milner et al., 1992; Jewell and McCourt, 2000).

Colent et al. (2000) were the first to show a modulation of pseudoneglect following prism adaptation: after adaptation to a leftward optical deviation, pseudoneglect became a neglect-like behavior with a mental overrepresentation of the right part of space and an underrepresentation of the left part of space. This representational aftereffect has been replicated many times in the lateral dimension (McCourt and Olafson, 1997; Berberovic and Mattingley, 2003; Michel et al., 2003; Fortis et al., 2011; Schintu et al., 2014, 2017; Michel and Cruz, 2015).

Spatial representation is not restricted to spatial representation (e.g., visual stimuli) but it extends to representation of spatially valued elements. For instance, in the visual modality, numbers have been demonstrated to be spatially represented along a mental horizontal line: small numbers (e.g., 1, 2, 3) are associated with the left part of the line, and large numbers (e.g., 18, 19, 20) are associated with the right part of the line (Dehaene et al., 1993). When healthy participants have to estimate the center between two numbers, they show a pseudoneglect bias toward the smaller numbers (i.e., stimuli spatially represented in the left part of space; Longo and Lourenco, 2007; Loftus et al., 2008, 2009). Similar to the line-bisection task, prism adaptation to a leftward optical deviation shifts this numerical bias toward the larger numbers (i.e., represented in the right part of space; Loftus et al., 2008). In the auditory modality, frequencies are also spatially represented along a mental horizontal line and along a mental vertical line: low auditory frequencies are associated with the left and the lower parts of space, and high auditory frequencies are associated with the right and the upper parts of space (Rusconi et al., 2006; Lidji et al., 2007; Ishihara et al., 2013). Musical expertise also influences the spatial association of auditory frequencies: horizontal and vertical associations are automatic for musicians, whereas the vertical association is rather a privilege of non-musicians. The horizontal association of auditory frequencies is present in non-musicians in an explicit condition exclusively (i.e., a task with simple pure tones and explicit instruction; Lidji et al., 2007). Recently, two studies demonstrated the aftereffects of prism adaptation in auditory frequency representation (Michel et al., 2019; Bonnet et al., 2021). These authors used the auditory interval bisection judgment task in which participants had to judge if a pure tone is closer to the low or the high limit of an auditory interval. Initially, the participants presented an auditory pseudoneglect toward lower auditory frequencies, which was shifted toward higher auditory frequencies after prism adaptation to a leftward optical deviation. Even if these results occurred regardless of musical expertise aftereffects were more marked in musicians (Bonnet et al., 2021). This contrast would be due to greater ease for musicians to horizontally represent auditory frequencies in relation to non-musicians who favor a vertical representation of auditory frequencies.

To the best of our knowledge, investigations on vertical prism adaptation are rare. The oldest research studies as well as those carried out in the last 20 years aimed to study mainly the lateral dimension. Consequently, the majority of studies showed sensorimotor and cognitive aftereffects of lateral prism adaptation of healthy subjects (for a review, see Michel, 2016) and neglect patients (for a review, see Jacquin-Courtois et al., 2013). At a representational level (i.e., without prism adaptation), studies that investigated vertical line-bisection task showed an initial bias above the veridical center toward the upper part of space (e.g., Drain and Reuter-Lorenz, 1996; McCourt and Olafson, 1997; Fink et al., 2001; Suavansri et al., 2012; Falchook et al., 2013; Churches et al., 2017; Chieffi et al., 2019). This bias that testifies to an overrepresentation of the upper part of space and an underrepresentation of the lower part of space is called altitudinal pseudoneglect. At a sensorimotor level, Bultitude et al. (2012) showed significant sensorimotor aftereffects only after vertical prism adaptation to an upward optical deviation. This present study then aimed to further investigate aftereffects of vertical prism adaptation on (1) sensorimotor performance (Experiments 1 and 2), (2) visual vertical space representation (Experiment 1), and (3) auditory frequency representation (Experiment 2).

First, if we consider symmetrical sensorimotor aftereffects following lateral prism adaptation, we assume that vertical prism adaptation to both upward and downward optical deviations produce sensorimotor aftereffects. However, based on the study of Bultitude et al. (2012) that showed significant sensorimotor aftereffects only after vertical prism adaptation to an upward optical deviation, it is also possible to observe asymmetrical sensorimotor aftereffects in this investigation.

Second, this study explored vertical visual space representation with a vertical line-bisection task on healthy participants, and the aftereffects of vertical prism adaptation in this representation. According to the literature, we expect to observe an initial upward estimation bias of the center of the vertical line (i.e., altitudinal pseudoneglect, e.g., Fink et al., 2001). With reference to the aftereffects of lateral prism adaptation on the lateral line-bisection task, we expect to observe asymmetrical aftereffects (i.e., only one optical deviation acts on vertical spatial representation), depending on the presence of pseudoneglect (Goedert et al., 2010). According to these observations, we predict a shift of the initial altitudinal bias toward the lower part of space following prism adaptation to an upward optical deviation.

Finally, this study investigated the aftereffects of vertical prism adaptation on auditory frequency representation. We expect to replicate the results of two recent studies, which showed auditory pseudoneglect toward lower auditory frequencies (Michel et al., 2019; Bonnet et al., 2021). As for the visual modality and because it was the case for lateral prism adaptation, we assume asymmetrical aftereffects of vertical prism adaptation on performance in auditory interval bisection judgments. More precisely, we hypothesize a shift of the initial auditory pseudoneglect toward higher auditory frequencies following prism adaptation to a downward optical deviation. We predict a dissociation of the aftereffects of vertical prism adaptation between the representation of the vertical visual space (i.e., aftereffect after adaptation to an upward optical deviation) and the representation of auditory frequencies (i.e., aftereffect after adaptation to a downward optical deviation).

## Experiment 1: Visuospatial Representation

All research procedures complied with this Declaration was adopted in Helsinki (Finland) in 1964 and were approved by the French Ethical Committee for the research in sports science (IRB00012476-2021-05-02-82).

### Materials and Methods

#### Sample Size Estimation

We conducted an *a priori* power analysis to define sample size estimation according to the data of the published study of Colent et al. (2000), *N* = 7 for each optical deviation, which is faithful to the main aim of the Experiment 1. The study compared the left and right responses collected with a horizontal Landmark task in pretest to those obtained in posttest after prism adaptation to a leftward or a rightward optical deviation. Based on mean subjective centers of the test phases for the leftward optical deviation (pretest: *M* = 123.98, SD = 1; posttest: *M* = 125.1, SD = 1.2), the effect size of this study was estimated to be *d* = 0.98. An *a priori* analysis for a dependent *t*-test comparison with α = 0.05 and power = 0.8 indicated a required sample size equal to *N* = 11 with the effect size mentioned above (G*Power; Faul et al., 2007). The current proposed sample sizes of *N* = 12 in one group and *N* = 11 in the other group are higher than the required sample size obtained by the *a priori* analysis.

#### Participants

Twenty-three healthy young adults participated in Experiment 1 (12 women, 11 men; age: 20–28 years; *M* = 22.65 years, SD = 2.5). They had normal or corrected-to-normal vision and, except for two ambidextrous, they were all right-handed (*M* = 0.7; SD = 0.3). Before beginning the experiment, each participant provided written consent after having received information notes. The participants were randomly allocated to prism adaptation to an upward or a downward optical deviation group (upward group: 12 participants; downward group: 11 participants). All the participants were naive to the purpose of the experiment and prism adaptation, and they were debriefed after the experiment.

#### Design and Experimental Setup

Each participant took part in a 1 h testing session, which was divided as follows: completion of questionnaires; preadaptation Landmark task; preadaptation visual open-loop pointing task; prism adaptation; postadaptation visual open-loop pointing task; postadaptation Landmark task; late-adaptation visual open-loop pointing task (to ensure that sensorimotor aftereffects were maintained until the end of the experiment).

**FIGURE 1:**
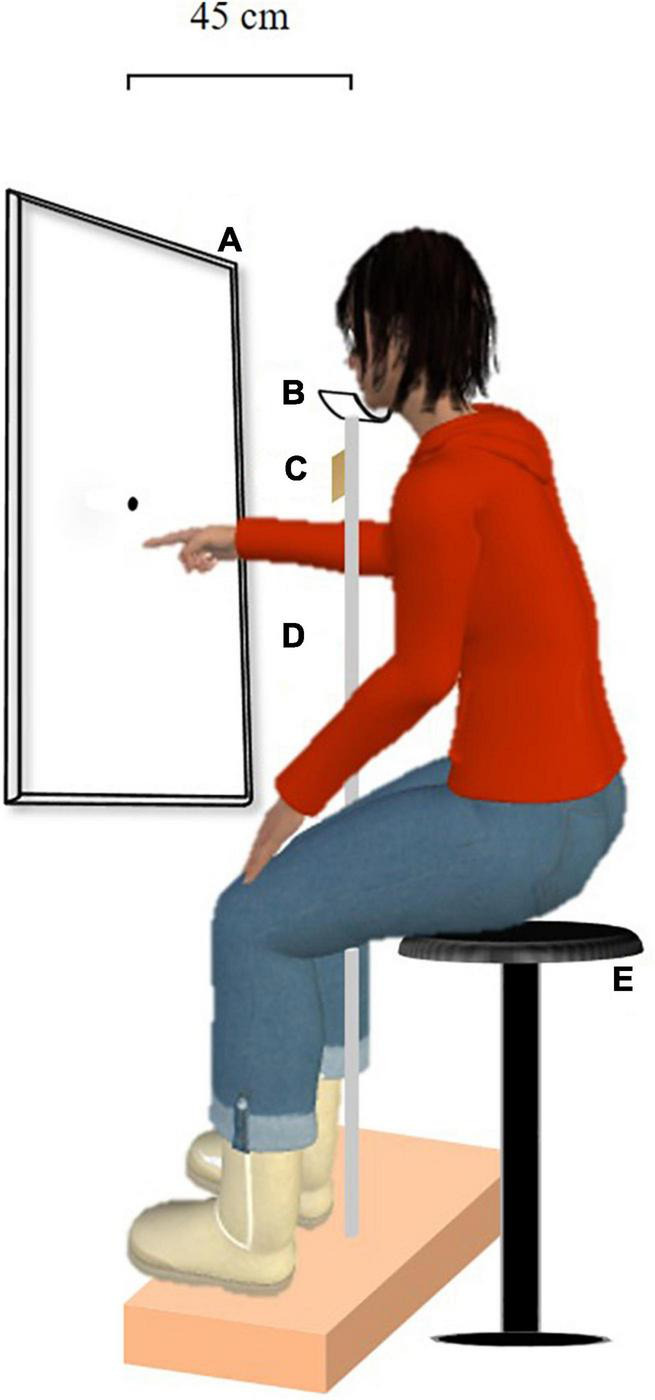
Experimental setup. The participants were seated on a stool adjustable in height facing a touchscreen whose center was aligned with their gaze throughout all test phases. The square bracket depicts the touchscreen-gaze distance of 45 cm. **(A)** Touchscreen, **(B)** chinrest, **(C)** wavy surface, **(D)** chin rest stem, and **(E)** stool adjustable in height.

The participants were seated facing a touchscreen (length: 924 mm, width: 520 mm) on a stool adjustable in height at a viewing distance of 45 cm (see Figure 1). They kept their head in chin rest to ensure that gaze was aligned with the touchscreen center throughout all test phases. During the Landmark task, the participants kept their hands on their knees. During the pointing task, the experimenter placed the participants’ right index either on the upper end of the chin rest stem (visual open-loop pointing task) or on the wavy surface laterally under the chin rest (prism adaptation); in both cases, the initial position of the right hand was never visible to the participants.

#### Landmark Task

The participants performed a perceptual line-bisection task before and after prism adaptation. A series of vertical black lines against a white background was displayed centrally on the touchscreen, and the center of the line was aligned with the participants’ gaze in their median sagittal plane. Each line was 250-mm long and 1-mm thick, and bisected by a 4-mm horizontal line, 1-mm thick, at either the true center or 2, 4, 6, 8, 10, or 12 mm above or below the true center (i.e., 13 bisections). For each bisected line, the participants had to indicate if they perceived the bisection as higher or lower than the true center of the line. They could not say that the bisection was in the line center, even if they felt that it was the case (i.e., forced-choice judgment). They orally gave their responses that were recorded by the experimenter. The lines appeared successively, and a plain anthracite gray screen was displayed between each line to avoid interference effects from one line to the other. Each bisected line was presented eight times across eight blocks (i.e., 104 stimuli) in a pseudorandomized order (i.e., not two consecutive same bisected lines). Because decreased alertness can bias line-bisection performance, as has been observed in lateral dimension (Dufour et al., 2007), the participants took a break for a few seconds between each block.

#### Visual Open-Loop Pointing Task

To check the effective development of adaptation, sensorimotor aftereffects were measured using the visual open-loop pointing task (i.e., without visual control during movement execution). The task was built as a vertical equivalent of the standard visuomanual open-loop pointing task in the horizontal plane (e.g., Colent et al., 2000). The participants were asked to look at the single sagittal visual target (black dot, diameter: 6 mm) displayed on the touchscreen, and then to point to this target by keeping their eyes closed. To avoid deadaptation, the experimenter passively placed the participants’ right index on the starting position before each trial, and the participants kept their eyes closed. Ten trials were performed before prism adaptation, immediately after prism removal, and after the completion of the postadaptation Landmark task.

#### Prism Adaptation

The vertical prism adaptation task was conceived based on the classical procedure used in the horizontal orientation (e.g., Rossetti et al., 1998; Berberovic and Mattingley, 2003). Immediately following the preadaptation visual open-loop pointing task, the participants wore optical wedge-prisms, which vertically displaced the visual field by 15° upward (i.e., base-down lenses) or downward (i.e., base-up lenses). Nine colored visual targets (diameter: 6 mm; interdot spaces: 4 cm) were vertically displayed on the vertical touchscreen, with the central target matched with the center of the touchscreen, aligned 45 cm from the starting point, and with the participants’ eyes. Four targets were placed on each side of the central target. For approximately 17 min, the experimenter told the participants which target to point at as quickly and as accurately as possible. The pointing movements were performed in blocks (i.e., four blocks of 81 pointing movements each for a total of 324 pointing movements); each block was composed of random pointing of all the targets, orally indicated by the experimenter. At the end of each movement, the participants returned their right index to the starting position (see Figure 1C: wavy surface). To ensure the optimal development of adaptation, vision of the starting position of the hand was occluded (Redding and Wallace, 1997). When the prism adaptation phase was achieved, the participants closed their eyes, and the experimenter removed the prism goggles.

#### Data Analyses

##### Visual Open-Loop Pointing Task

To appraise sensorimotor aftereffects, angular pointing errors from the sagittal target were measured in degrees: downward errors were expressed in negative values and upward errors in positive values. In order to compare the magnitude of sensorimotor aftereffects between both optical deviations, we computed the absolute value of the difference between posttest and pretest (i.e., posttest–pretest; immediate aftereffects), and between late-test and pretest (i.e., late-test–pretest; late after effects).

##### Landmark Task

In the Landmark task, we computed the percentage of trials on which the participants indicated that the transector was below the perceived line midpoint. This percentage of “low” responses provides the proportion of transectors perceived as located in the lower part of the line and gives an approximate estimation of the visual subjective center. A weak percentage of “low” responses corresponds to a subjective visual center lower than the objective visual center; in contrast, a large percentage of “low” responses corresponds to a subjective visual center higher than the objective visual center. The second computed parameter was the point of subjective equality precisely defined by fitting the data with a sigmoid function. This subjective visual center of the vertical line is the line location for which the participants provided 50% of “low” responses and 50% of “high” responses.

#### Statistical Analysis

In each experiment, separate statistical analyses were carried out for each optical deviation in order to investigate aftereffects of vertical prism adaptation according to the optical deviation. The analyses were achieved with the Statistica software (version 13.3), and effect sizes were computed with the JASP software (version 0.11.1); the threshold for statistical significance was set to α = 0.05. Because the data followed the normal distribution (Shapiro–Wilk: all *p*s > 0.05), we conducted parametric tests. In order to assess a potential initial performance bias, we conducted a one-sample *t*-test to compare the data obtained in pretest to reference values (i.e., comparison to 0 for the visual open-loop pointing task; comparison to 50 for the percentage of “low” responses; comparison to the objective center for the subjective center). For the visual open-loop pointing task, a repeated measures ANOVA and, if necessary, Bonferroni *post hoc* comparisons were performed. For the Landmark task, we conducted *t*-tests to compare the posttest to the pretest for the percentage of “low” responses as well as the subjective center.

### Results

#### Visual Open-Loop Pointing Task: Sensorimotor AfterEffects

##### Adaptation to an Upward Optical Deviation

**FIGURE 2:**
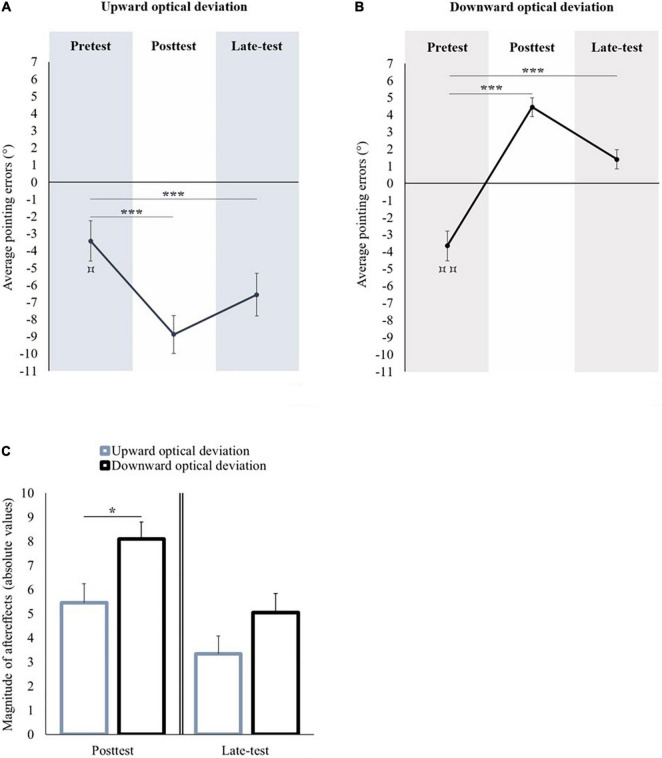
Sensorimotor aftereffects of vertical prism adaptation. Panel **(A)** displays the sensorimotor aftereffects before (pretest), immediately after (posttest) an upward optical deviation, and at the end of the experiment (late-test). Panel **(B)** displays the sensorimotor aftereffects before (pretest), immediately after (posttest) a downward optical deviation, and at the end of the experiment (late-test). Panel **(C)** displays the absolute values of sensorimotor aftereffects occurred following upward and downward prism adaptations in the posttest (left part), and in the late-test (right part). *p < 0.05; ***p < 0.001. Comparison of the pretest to 0: ^⌑^*p* < 0.05; ^⌑⌑^*p* < 0.01.

In the pretest, the average pointing error was significantly lower than the midsagittal visual target [*t*(11) = −2.934, *p* = 0.014, *d* = −0.847] (see Figure 2A). A repeated measures ANOVA with Session (pretest, posttest, and late-test) as within-subject factor showed a significant effect of Session [*F*(2,22) = 33.12, *p* < 0.001, η*_*p*_*^2^ = 0.751]. Compared to the pretest, the pointing performance was significantly shifted toward the lower part of space in the posttest (Bonferroni *post hoc* comparison: *p* < 0.001) and in the late-test (Bonferroni *post hoc* comparison: *p* < 0.001). All the participants remained adapted until the end of the experiment.

##### Adaptation to a Downward Optical Deviation

In the pretest, the average pointing error was significantly lower than the midsagittal visual target [*t*(10) = −4.181, *p* = 0.002, *d* = −1.261] (see Figure 2B). A repeated measures ANOVA with Session (pretest, posttest, and late-test) as within-subject factor showed a significant effect of Session [*F*(2,20) = 82.104, *p* < 0.001, η*_*p*_*^2^ = 0.891]. Compared to the pretest, the pointing performance was significantly shifted toward the upper part of space in the posttest (Bonferroni *post hoc* comparison: *p* < 0.001) and in the late-test (Bonferroni *post hoc* comparison: *p* < 0.001). All the participants remained adapted until the end of the experiment.

##### Magnitude of Sensorimotor AfterEffects: Upward Versus Downward Optical Deviation

The performance in the pretest did not differ between both groups of optical deviation [*t*(21) = 0.148; *p* = 0.884]. The magnitude of the aftereffects in the posttest was significantly higher after prism adaptation to a downward optical deviation in comparison to prism adaptation to an upward optical deviation [*t*(21) = −2.542, *p* = 0.019, *d* = 1.061]. In the late-test, the magnitude of aftereffects did not significantly differ between both optical deviations [*t*(21) = −1.636, *p* = 0.117] (see Figure 2C).

#### Landmark Task: Cognitive Aftereffects

##### Comparison in Pretest Between Both Groups of Optical Deviation

###### Percentages of “Low” Responses

In the pretest, the mean percentages of “low” responses did not significantly differ between both groups of optical deviation [*t*(21) = −1.321, *p* = 0.201].

###### Subjective Visual Center

In the pretest, the mean subjective visual center was not significantly different between both groups of optical deviation [*t*(21) = −1.125, *p* = 0.273].

##### Adaptation to an Upward Optical Deviation

###### Percentages of “Low” Responses

**FIGURE 3:**
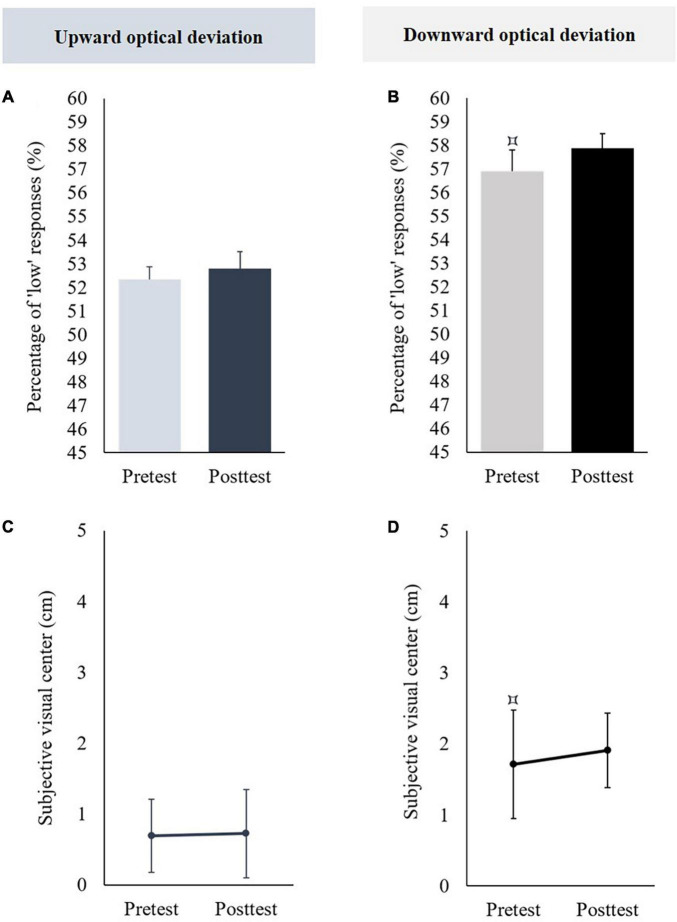
Aftereffects of vertical prism adaptation on visuospatial representation. Data obtained in the group using an upward optical deviation are represented in the left part; data obtained in the group using a downward prism adaptation are represented in the right part. Panels **(A,B)** display the percentage of “low” responses before (pretest) and after (posttest) vertical prism adaptation; panels **(C,D)** display the subjective visual center before (pretest) and after (posttest) vertical prism adaptation. The pretest was compared to 50 [for the percentage of “low” responses, panels **(A,B)**] or 0 [i.e., objective visual center, panels **(C,D)**]: ^⌑^*p* < 0.05.

In the pretest, the mean percentages of “low” responses did not significantly differ from 50% [*t*(11) = 1.203, *p* = 0.254]. When the posttest was compared to the pretest, no significant difference was observed [*t*(11) = −0.295, *p* = 0.773] (see Figure 3A).

###### Subjective Visual Center

The mean subjective visual center was not significantly different from 0 in the pretest [*t*(11) = 1.336, *p* = 0.208]. When the posttest was compared to the pretest, no significant difference was observed [*t*(11) = −0.088, *p* = 0.931] (see Figure 3C).

##### Adaptation to a Downward Optical Deviation

###### Percentages of “Low” Responses

In pretest, the mean percentages of “low” responses were significantly higher than 50% [*t*(10) = 2.341, *p* = 0.041, *d* = 0.706]. When the posttest was compared to the pretest, no significant difference was observed [*t*(10) = −0.432, *p* = 0.675] (see Figure 3B).

###### Subjective Visual Center

The mean subjective visual center was significantly higher than 0 in the pretest [*t*(10) = 2.241, *p* = 0.049, *d* = 0.676]. When the posttest was compared to the pretest, no significant difference was observed [*t*(10) = −0.334, *p* = 0.745] (see Figure 3D).

### Discussion of Experiment 1

First, an initial sensorimotor bias directed toward the lower part of space was observed in most of the participants who made downward pointing errors during the visual open-loop pointing task (i.e., without visual feedback). Vertical prism adaptation to an upward optical deviation exacerbated this initial sensorimotor bias, whereas vertical prism adaptation to a downward optical deviation shifted the initial bias toward the higher part of space. For the first time, we demonstrated sensorimotor aftereffects after vertical prism adaptation to both optical deviations, as it is usually observed after lateral prism adaptation.

Then, an initial representational bias directed toward the higher part of the visual space was observed during the Landmark task. Named altitudinal bias, this result is consistent with previous studies, which investigated vertical visual representation by means of a Landmark task (McCourt and Olafson, 1997; Fink et al., 2001).

Finally, adaptation to a vertical prism did not affect the visuospatial representation, regardless of the optical deviation used. This result differs from those observed after lateral prism adaptation to a leftward optical deviation, which shifted the subjective center of healthy participants toward the right part of space (e.g., Colent et al., 2000; Berberovic and Mattingley, 2003; Michel et al., 2003; Michel, 2016).

Altogether, the results of Experiment 1 were the first demonstration of sensorimotor aftereffects after vertical prism adaptation to both optical deviations. We failed to observe vertical prism adaptation aftereffects on visuospatial representation. However, before concluding on an absence of cognitive aftereffects of vertical prism adaptation, it is necessary to assess whether vertical prism adaptation aftereffects can be observed on spatially valued elements in other modalities, which, as visuospatial representation, have been also shown to be sensitive to lateral prism adaptation. In Experiment 2, the Landmark task was replaced by an auditory interval bisection judgment to investigate auditory frequency representation and aftereffects of vertical prism adaptation on this representation.

## Experiment 2: Auditory Frequency Representation

All the research procedures complied with the Declaration of Helsinki (1964) and were approved by the French Ethical Committee for the research in sports science (IRB00012476-2021-05-02-82).

### Materials and Methods

The experimental procedure was similar to Experiment 1 (i.e., visual open-loop pointing task and prism adaptation) except for the Landmark task, which was replaced by auditory interval bisection judgment.

#### Sample Size Estimation

We conducted an *a priori* power analysis to define sample size estimation according to the data of the published study of Michel et al. (2019), *N* = 18 for each optical deviation, which is faithful to the main aim of Experiment 2. The study compared the low and high responses given in an auditory interval bisection judgment in the pretest and the posttest after prism adaptation to a leftward or rightward optical deviation. The effect size of this study was *d* = −1.08. An *a priori* analysis for a dependent *t*-test comparison with α = 0.05 and power = 0.80 indicated a required sample size equal to *N* = 9 with the effect size mentioned above (G*Power; Faul et al., 2007). The current proposed sample size of *N* = 12 in each group is higher than the required sample size obtained by the *a priori* analysis.

#### Participants

Twenty-four healthy young non-musician adults participated in Experiment 2 (14 women, 10 men; age: 18–31 years; *M* = 23.92 years, SD = 3.62). They had normal or corrected-to-normal vision and, except for two ambidextrous, they were all right-handed (*M* = 0.78; SD = 0.21). Before beginning the experiment, each participant provided written consent after having received information notes. The participants were randomly allocated to prism adaptation to an upward or downward optical deviation group (upward group: 12 participants; downward group: 12 participants). No participants participated in Experiment 1; they were naive to the purpose of the experiment and prism adaptation, and they were debriefed after the experiment.

#### Design and Experimental Setup

The design and experimental setup were similar to those of Experiment 1 for prism adaptation and the visual open-loop pointing task (see section “Design and Experimental Setup”).

#### Auditory Interval Bisection Judgment

The paradigm was the same as the one used by Michel et al. (2019). Auditory stimuli were pure tones created with the Amadeus Pro software and not related to the musical system; they were defined in Mel (i.e., perceptual unit) and converted to Hertz (i.e., physical unit; see Table 1). Two auditory frequencies, 724 and 1,330 Hz, defined the auditory interval for which the objective center was 1,027 Hz (see Table 1). Eleven other auditory frequencies (788; 884; 912; 941; 970; 1,000; 1,030; 1,061; 1,093; 1,125; and 1,242 Hz see Table 1) were used as target auditory frequencies (TAFs) in the auditory interval. In this auditory task, the participants had to orally indicate to the experimenter if the TAF was closer to the low or to the high limit of the auditory interval. The PsyScope software recorded all the responses and presented auditory stimuli to the participants with noise-isolating Sennheiser headphones (HD 202 model).

**TABLE 1:** Conversion table of auditory frequencies used in the auditory interval bisection judgment.

Auditory frequencies	Mel	Hertz (Hz)
Low limit of the auditory interval	800	724
Objective auditory center	1,018	1,027
High limit of the auditory interval	1,200	1,330
Target auditory frequencies	850; 920; 940; 960; 980; 1,000; 1,020; 1,040; 1,060; 1,080; 1,150	788; 884; 912; 941; 970; 1,000; 1,030; 1,061; 1,093; 1,125; 1,242

*The auditory frequencies were converted from the perceptual unit Mel to the physical unit Hertz according to the following mathematical formula: $2595\times \log\left(1+\frac{f}{700}\right)$.*

**FIGURE 4:**
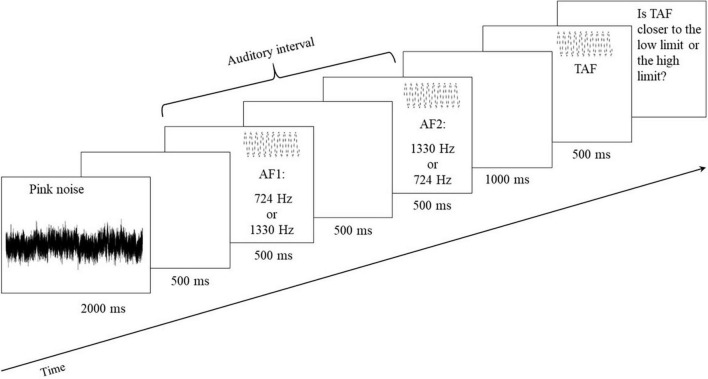
Event sequence for each trial of the auditory interval bisection judgment. One trial began with pink noise for 2,000 ms, followed by the presentation of auditory limits (i.e., AF1 and AF2; 500 ms), each separated by a silence of 500 ms. After the auditory interval, there was a silent period of 1,000 ms followed by the TAF (500 ms).

Every trial followed the event sequence displayed in Figure 4. In order to avoid auditory memory influences of the previous stimuli, each trial began with pink noise (2,000 ms). Then came a silent period of 500 ms followed by a presentation of the two auditory limits of the interval that lasted 500 ms each and were separated by a silence of 500 ms. For one-half of the trials, the first auditory frequency (AF1) was 724 Hz, and the second one (AF2) was 1,330 Hz, and vice versa for the second half of the trials. A silent interval of 1,000 ms followed the auditory interval presentation, and the TAF sounded for 500 ms. Each TAF was presented four times across two blocks, each composed of 22 stimuli: the same TAF was only heard twice per block without immediate repetition. This resulted in 44 trials, which were pseudorandomly ordered. For each participant and for both the pretest and posttest, there was a different random order of trials.

At the beginning of each trial, the participants carried out a training in which they had to differentiate the two auditory limits (i.e., 724 and 1,330 Hz), and they performed four training trials of the auditory interval bisection judgment. All the participants reported no difficulty in achieving the training; they reported to have clearly understood the instructions. Overall, the auditory task lasted between 12 and 15 min.

#### Data Analyses

##### Visual Open-Loop Pointing Task

The analysis of sensorimotor data was similar to Experiment 1 (see section “Open-Loop Pointing Task”).

##### Auditory Interval Bisection Judgment

The analysis of data from the auditory interval bisection judgment was similar to that of the Landmark task. The mean percentage of “low” responses was computed. It indicates the proportion of TAF considered as closer to the low auditory limit (i.e., 724 Hz; see Table 1), and it gives an approximate estimation of the subjective auditory interval center. A weak percentage of “low” responses corresponds to a subjective auditory center lower than the objective auditory center (i.e., 1,027 Hz; see Table 1); this would suggest an auditory bias of the estimated interval center toward the lower auditory frequencies. In contrast, a large percentage of “low” responses corresponds to a subjective auditory center higher than the objective auditory center (i.e., 1,027 Hz; see Table 1); this would suggest an auditory bias of the estimated interval center toward the higher auditory frequencies. The second computed parameter was the point of subjective equality precisely defined by fitting the data with a sigmoid function. This subjective auditory center of the auditory interval is the frequency for which the participants provided 50% of “low” responses and 50% of “high” responses (Michel et al., 2019).

#### Statistical Analysis

The statistical analyses were similar to Experiment 1 (see section “Statistical Analysis”).

### Results

#### Visual Open-Loop Pointing Task: Sensorimotor Aftereffects

##### Adaptation to an Upward Optical Deviation

**FIGURE 5:**
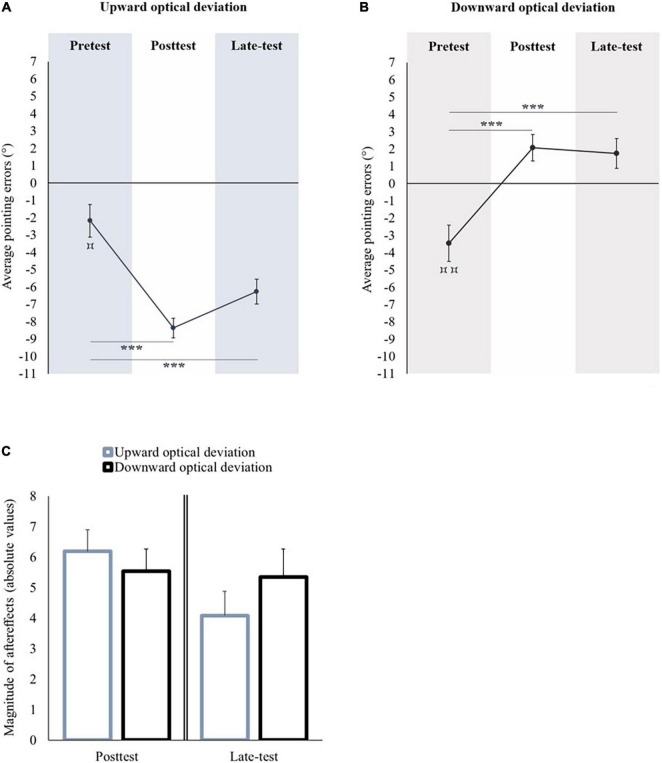
Sensorimotor aftereffects of vertical prism adaptation. Panel **(A)** displays sensorimotor aftereffects before (pretest), immediately after (posttest) an upward optical deviation, and at the end of the experiment (late-test). Panel **(B)** displays the sensorimotor aftereffects before (pretest), immediately after (posttest) a downward optical deviation, and at the end of the experiment (late-test). Panel **(C)** displays the absolute values of sensorimotor aftereffects occurred following upward and downward prism adaptations in the posttest (left part) and in the late-test (right part). ***p < 0.001. Comparison of the pretest to 0: ^⌑^*p* < 0.05; ^⌑⌑^*p* < 0.01.

In the pretest, the participants pointed significantly lower than the midsagittal visual target [*t*(11) = −2.346, *p* = 0.039, *d* = −0.677]. A repeated measures ANOVA with Session (pretest, posttest, and late-test) as within-subject factor showed a significant effect of Session [*F*(2,22) = 46.017, *p* < 0.001, η*_*p*_*^2^ = 0.807]. The initial bias was significantly exacerbated toward the lower part of space in the posttest (Bonferroni *post hoc* comparison: *p* < 0.001) and in the late-test (Bonferroni *post hoc* comparison: *p* < 0.001) (see Figure 5A).

##### Adaptation to a Downward Optical Deviation

In the pretest, the participants pointed significantly lower than the midsagittal visual target [*t*(11) = −3.312, *p* = 0.007, *d* = −0.956]. A repeated measures ANOVA with Session (pretest, posttest, and late-test) as within-subject factor showed a significant effect of Session [*F*(2,22) = 32.933, *p* < 0.001, η*_*p*_*^2^ = 0.75]. The initial bias was significantly shifted toward the upper part of space in the posttest (Bonferroni *post hoc* comparison: *p* < 0.001) and in the late-test (Bonferroni *post hoc* comparison: *p* < 0.001) (see Figure 5B).

##### Magnitude of Sensorimotor AfterEffects: Upward Versus Downward Optical Deviation

The performance in the pretest did not differ between both groups of optical deviation [*t*(22) = 0.91, *p* = 0.373]. No significant difference was observed between both optical deviations, either in the posttest [*t*(22) = 0.64, *p* = 0.529] or in the late-test [*t*(22) = −1.034, *p* = 0.313] (see Figure 5C).

#### Auditory Interval Bisection Judgment: Cognitive AfterEffects

##### Comparison of Pretests Between Both Experimental Groups

###### Percentages of “Low” Responses

In the pretest, the mean percentages of “low” responses did not significantly differ between both groups of optical deviation [*t*(22) = −0.405, *p* = 0.69].

###### Subjective Auditory Center

In the pretest, the mean subjective visual center was not significantly different between both groups of optical deviation [*t*(22) = −0.194, *p* = 0.848].

##### Adaptation to an Upward Optical Deviation

###### Percentages of “Low” Responses

**FIGURE 6:**
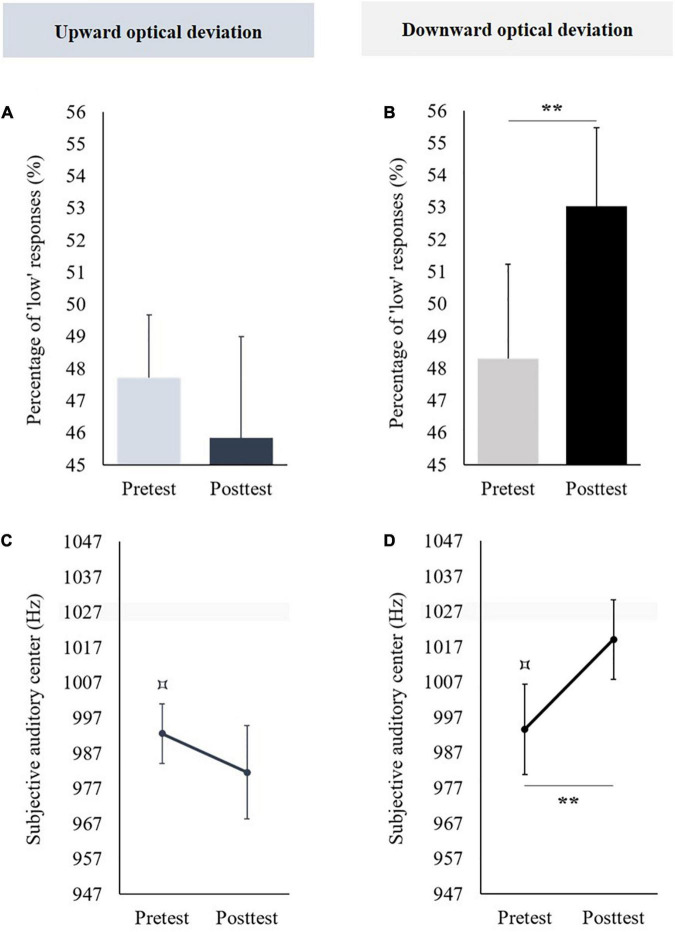
Aftereffects of vertical prism adaptation on auditory frequency representation. Data obtained in the group using an upward optical deviation are represented in the left part; data obtained in the group using a downward optical deviation are represented in the right part. Panels **(A,B)** display the percentage of “low” responses before (pretest) and after (posttest) vertical prism adaptation. Panels **(C,D)** display the subjective visual center before (pretest) and after (posttest) vertical prism adaptation. **p < 0.01. The pretest was compared to 50 % [for the percentage of “low” responses, panels **(A,B)**] or 1,027 Hz [i.e., the objective auditory center, panels **(C,D)**]: ^⌑^*p* < 0.05.

In the pretest, the mean percentage of “low” responses was not significantly different from 50% [*t*(11) = −1.173, *p* = 0.266]. Prism adaptation to an upward optical deviation did not produce significant aftereffects on percentages of “low” responses [*t*(11) = 0.649, *p* = 0.53] (see Figure 6A).

###### Subjective Auditory Center

In the pretest, the mean subjective auditory center was significantly lower than the objective auditory center (i.e., 1,027 Hz) [*t*(11) = −4.08, *p* = 0.002, *d* = −1.178]. Prism adaptation to an upward optical deviation did not significantly change the subjective auditory center [*t*(11) = 0.91, *p* = 0.382] (see Figure 6C).

##### Adaptation to a Downward Optical Deviation

###### Percentages of “Low” Responses

In the pretest, the mean percentage of “low” responses was not significantly different from 50% [*t*(11) = −0.581, *p* = 0.573]. Prism adaptation to a downward optical deviation significantly increased the percentages of “low” responses [*t*(11) = −3.654, *p* = 0.004, *d* = −1.055] (see Figure 6B).

###### Subjective Auditory Center

In the pretest, the mean subjective auditory center was significantly lower than the objective auditory center (i.e., 1,027 Hz) [*t*(11) = −2.617, *p* = 0.024, *d* = −0.755]. Prism adaptation to a downward optical deviation significantly increased the subjective auditory center [*t*(11) = −3.322, *p* = 0.007, *d* = −0.959] (see Figure 6D).

### Discussion of Experiment 2

As was the case in Experiment 1, an initial sensorimotor bias toward the lower part of space was observed in most of the participants, which was affected after vertical prism adaptation. These results confirm those observed in Experiment 1. Unlike in Experiment 1, sensorimotor adaptation was of the same magnitude in Experiment 2 regardless of the optical deviation used.

Then, a significant initial bias was observed for the auditory subjective center but not for the percentage of “low” responses, even if the percentage was lower than 50% in the pretest. Most of the participants estimated the center of the auditory interval lower than the objective auditory center; in other words, the subjective auditory center was initially shifted toward low auditory frequencies (i.e., auditory frequencies associated with the lower part of space). Named auditory frequency pseudoneglect, this initial auditory representational bias is in accordance with observations of previous studies (Michel et al., 2019; Bonnet et al., 2021).

Finally, only adaptation to a downward optical deviation changed the auditory frequency representation. In the posttest, the auditory subjective center was shifted toward the higher auditory frequencies, which are spatially associated with the higher part of space. This result is similar to those observed after lateral prism adaptation to a leftward optical deviation, which shifted the auditory subjective center toward higher auditory frequencies, which are also spatially associated with the right part of space (Michel et al., 2019; Bonnet et al., 2021).

Altogether, the results of Experiments 1 and 2 supported the existence of an initial sensorimotor bias directed toward the lower part of space and confirmed the development of sensorimotor aftereffects after vertical prism adaptation to both optical deviations. Furthermore, Experiment 2 approved (1) the presence of an initial auditory pseudoneglect toward lower auditory frequencies, which (2) was shifted only after adaptation to a downward optical deviation.

## General Discussion

The main objectives of this study were to (1) assess the sensorimotor aftereffects of vertical prism adaptation, (2) investigate vertical space representation and auditory frequency representation, and (3) evaluate the cognitive aftereffects of vertical prism adaptation on these representations. In Experiments 1 and 2, most of the participants made pointing movements below the actual position of the visual target in the pretest. Prism adaptation to an upward optical deviation exacerbated this initial sensorimotor bias toward the lower part of space, whereas prism adaptation to a downward optical deviation shifted this initial sensorimotor bias toward the higher part of space. These results were the first demonstration of sensorimotor aftereffects after vertical prism adaptation to both optical deviations. Concerning visual space representation, Experiment 1 showed an initial altitudinal spatial bias toward the higher part of space, which was unchanged after vertical prism adaptation. On the other hand, Experiment 2 showed an auditory pseudoneglect directed toward lower auditory frequencies in the pretest, which was shifted toward higher auditory frequencies after vertical prism adaptation to a downward optical deviation. These innovative asymmetrical cognitive aftereffects of vertical prism adaptation were the first to be observed in auditory frequency representation.

### Vertical Prism Adaptation Produces Sensorimotor AfterEffects Following Both Optical Deviations

Gravity could explain the initial sensorimotor bias observed in most of the participants of this study: being a downwardly attractive force (for a review: White et al., 2020), it may have naturally moved the participants’ arm toward the lower part of space during the visual open-loop pointing task in the pretest.

Innovative sensorimotor aftereffects were observed following vertical prism adaptation to both optical deviations; they were replicated in both Experiments 1 and 2. Sensorimotor aftereffects of vertical prism adaptation are substantially similar to those observed after lateral prism adaptation (e.g., Redding and Wallace, 2006): prism adaptation produces sensorimotor aftereffects in the opposite direction of the optical deviation used, in both horizontal and vertical spatial dimensions. Our results are in accordance with those of Martin et al. (2001) who assessed the performance of healthy participants in ball-throwing toward a visual target before, during, and after vertical prism adaptation to a downward optical deviation. They showed sensorimotor aftereffects after a downward prism adaptation: the final position of the ball was shifted toward the higher part of space (Martin et al., 2001). Even if this result supports the outcomes of this study, data concerning vertical prism adaptation to an upward optical deviation are missing in the study of Martin et al. (2001). Bultitude et al. (2012) used subjective straight-ahead pointing in open-loop conditions to investigate sensorimotor aftereffects after vertical prism adaptation to both optical deviations in healthy old people. In contrast to our results and those of Martin et al. (2001), exhibiting upward aftereffects after adaptation to a downward optical deviation, the authors did not show aftereffects after adaptation to a downward optical deviation. To explain the absence of sensorimotor after-effect development in the downward-shifting prism group, Bultitude et al. suggested more prevalent fatigue for upward aftereffects because of the need to fight against gravity. The resulting fatigue would have shifted the trajectory of the adapted arm downward, counteracting the upward aftereffects after adaptation to a downward optical deviation and, perhaps, boosting the downward aftereffects of adaptation to an upward optical deviation (Bultitude et al., 2012).

### Vertical Prism Adaptation Does Not Modify the Visuospatial Representation

An initial bias was observed in the Landmark task in the group exposed to a downward optical deviation: in the pretest, the percentage of “low” responses was higher than 50%, and the participants estimated the center of the line (i.e., subjective center) higher than the real one (i.e., objective center). A similar initial bias was observed in the group exposed to an upward optical deviation, but it failed to reach significance. This representational bias was already shown in previous studies, which investigated manual line-bisection tasks (Suavansri et al., 2012; Falchook et al., 2013; Churches et al., 2017; Chieffi et al., 2019) or perceptual line-bisection tasks (i.e., Landmark task; McCourt and Olafson, 1997; Fink et al., 2001). The upward bias would depend on object-based attention, because it has been shown to vary according to the shape of the stimulus to be bisected (Churches et al., 2017) and to task instructions (i.e., comparison of line length versus center line estimation; Drain and Reuter-Lorenz, 1996). In the same vein, the upward bias increased when famous faces, which request object-based attention, had to be remembered during the vertical line-bisection task (Claunch et al., 2012). More precisely, the upward bias appeared to be more marked when participants employed an object-based representational strategy. It has been shown that object-based attention is mediated by the visual ventral stream (Goodale and Milner, 1992). Cerebral lesions in this ventral stream (i.e., bilateral inferior occipitotemporal) led to upper visual field neglect (Mennemeier et al., 1992), and they decreased altitudinal attentional bias (Hromas et al., 2020). Consequently, the upward bias observed in this study can be explained by the activation of the visual ventral stream during the vertical Landmark task (Drain and Reuter-Lorenz, 1996; Mańkowska et al., 2021). Moreover, in a more ecological way, the visual attention is directed toward the upper part of the objects, because it contains the most useful information on what the object is (Churches et al., 2017).

Vertical prism adaptation did not change the performance of healthy participants in the vertical Landmark task, regardless of the optical deviation used. These results were surprising because, according to the literature having investigated lateral prism adaptation, we assumed aftereffects of vertical prism adaptation on visuospatial representation. Indeed, prism adaptation to a leftward optical deviation shifts the initial leftward bias toward the right part of space, which led us to expect a shift of the initial upward bias toward the lower part of space after an upward prism adaptation.

In this study, the absence of aftereffects on the Landmark task following vertical prism adaptation could be explained by at least two factors. First, because aftereffects depend on the presence of pseudoneglect (Goedert et al., 2010), the absence of aftereffect in the group submitted to an upward prism adaptation could be explained by the absence of a significant altitudinal pseudoneglect in the pretest. Furthermore, if we refer to the literature on lateral dimension showing representational aftereffects only after adaptation to an optical deviation in the direction of the baseline representational bias (Goedert et al., 2010), the presence of a significant upward bias in the group exposed to a downward optical deviation could not be shifted. Second, the reason could be methodological: the Landmark task was not sensitive enough to observe cognitive aftereffects following vertical prism adaptation. Perhaps, a timeout should be imposed on participants in order to limit the reflection period. Moreover, the manual version of the line-bisection task could be more sensitive than the perceptual version, because it could involve not only attentional bias but also intentional bias. The use of the right hand to perform the manual line-bisection task has been shown to produce a greater upward bias (Suavansri et al., 2012). It has been suggested that lateral prism adaptation seems to have more influence in tasks requiring a manual response in comparison with perceptual tasks such as the Landmark (Striemer et al., 2016). Consequently, the use of the right hand during the line-bisection task could increase the initial upward bias as well as its shift toward the lower part of space after upward prism adaptation. Another perspective would be to evaluate whether prism adaptation could modulate an intentional bias response by using the LANDMARK-V task before and after prism adaptation. In this verbal response task, in which one of the segments of the line is black and the other is red, participants had to name the color of the longer (or the shorter) segment (Bisiach et al., 1998a).

### Vertical Prism Adaptation Changes Auditory Frequency Representation in Non-musicians

In both groups of optical deviation, an initial bias was observed for the subjective auditory center but not for the percentage of “low” responses. In the pretest, the participants estimated the center of the auditory interval lower than the real center; in other words, the subjective auditory center was initially shifted toward lower auditory frequencies, which are associated with the lower part of space.

This initial bias is consistent with the one observed in previous studies (Michel et al., 2019; Bonnet et al., 2021). Adaptation to a downward optical deviation increased the percentage of “low” responses and shifted the initial bias toward higher auditory frequencies (i.e., auditory frequencies spatially represented in the higher part of space). These results conformed with those observed in previous studies, which investigated aftereffects on auditory spatial representation after lateral prism adaptation to a leftward optical deviation (Michel et al., 2019; Bonnet et al., 2021). Aftereffects of vertical prism adaptation on auditory frequency, representation seems to be more pronounced than those following lateral prism adaptation. Michel et al. (2019) did not show auditory aftereffects following lateral prism adaptation in non-musicians, whereas auditory aftereffects were significant following vertical prism adaptation in this study. The greatest facility for vertical prism adaptation to modify the subjective auditory center and percentage of “low” responses could be explained by the fact that non-musicians seem to favor a vertical auditory frequency representation rather than a horizontal one (Rusconi et al., 2006). Our study provides new evidence for crossmodal cognitive aftereffects of prism adaptation, which acts on high-order cognitive functions and sensorial modalities not directly involved during prism adaptation.

### Vertical Versus Lateral Prism Adaptation: Same Involvement of Neural Processes?

This study testified to the crossmodal cognitive aftereffects of vertical prism adaptation. Prism adaptation to a downward optical deviation increased the subjective auditory center toward higher auditory frequencies, associated with the right and the upper parts of space, as was the case after lateral prism adaptation to a leftward optical deviation (Bonnet et al., 2021). However, vertical prism adaptation did not change visuospatial representation, whereas a shift of the subjective visual center occurred after leftward prism adaptation in the healthy participants (for reviews: Michel, 2016; McIntosh et al., 2019).

The current lack of aftereffects in the visuospatial representation may be due to the less-marked hemispheric lateralization in vertical dimension processing. The vertical dimension would involve neuroanatomical substrates more bilaterally than the lateral dimension, even if both dimensions involve right hemisphere dominance. Indeed, it has been shown that in healthy individuals, vertical lines activated the superior parietal posterior cortex and the medial striate and extrastriate cortex bilaterally, whereas horizontal lines activated the right lateral striate and extrastriate cortex (Fink et al., 2001). Moreover, two recent case studies demonstrated altitudinal neglect following cerebral impairments: bilateral parietal atrophy (i.e., dorsal visual stream) led to neglect of the lower visual field (Julayanont et al., 2019), whereas a lesion in the right temporal lobe (i.e., ventral visual stream) caused neglect of the higher visual field (Morris et al., 2020). Weaker hemispheric lateralization of the vertical dimension could make it difficult to observe cognitive aftereffects of prism adaptation in this orientation, in comparison with the lateral dimension.

Unlike vertical visual lines, auditory frequency perception involves a more lateralized neural substrate. The right hemisphere is dominant in auditory perception (e.g., Hyde et al., 2008) and in pitch discrimination (Liégeois-chauvel et al., 2001; Zatorre and Belin, 2001), more precisely in the superior temporal gyrus including the planum temporale and especially the Heschl gyrus, which is considered as the “pitch center” (Hall and Plack, 2009). The asymmetry in favor of the right hemisphere in pitch encoding also involves activation of the right inferior frontal gyrus (Hyde et al., 2006; for a review, see Griffiths and Hall, 2012). Moreover, the parietal cortex comprises multimodal neurons (Stein and Stanford, 2008), and it is activated during tasks that use auditory or visual stimuli, especially in the lateral intraparietal area (e.g., Cohen, 2009). According to these investigations of dynamic changes in brain activity during pitch processing and auditory mental representation, associated with our results showing cognitive aftereffects of vertical prism adaptation on auditory frequency mental representation, vertical prism adaptation could act on the right hemisphere. This assumption coincides with the attentional model proposed by Clarke and Crottaz-herbette (2016) in which the superior temporal gyrus is mobilized in the ventral attentional system. Thus, cognitive aftereffects of vertical prism adaptation were observed on auditory frequency representation probably because vertical prism adaptation would act on the right hemisphere, which is more activated in both pitch discrimination and auditory mental representation.

The issue addressed in this study is new and innovative. Thus, the existing literature studying this thematic is very scarce and makes the interpretation of the results, as well as the identification of neural bases of vertical prism adaptation, difficult and limited. Nevertheless, the present new results suggest (1) that both vertical and lateral prism adaptations act on substrates activated during pitch processing, especially in the right hemisphere, and (2) that the more bilateral cerebral involvement of vertical visuospatial representation could prevent the development of cognitive aftereffects of vertical prism adaptation on this modality.

## Conclusion

Our study provides new behavioral results about the aftereffects of vertical prism adaptation. For the first time, sensorimotor aftereffects occurred after upward and downward prism adaptations, in the opposite direction of the optical deviation. At a sensorimotor level, prism adaptation would seem to behave in the same way in the vertical and lateral dimensions. Unlike lateral prism adaptation cognitive aftereffects that have been previously shown in both visuospatial horizontal representation and auditory frequency representation, cognitive aftereffects of vertical prism adaptation occurred in the auditory frequency representation but not in the vertical visuospatial representation. These results suggest that both vertical and lateral prism adaptations share a common substrate dedicated to the auditory modality (probably the temporal cortex), and that vertical adaptation does not act on the neural substrate of vertical visuospatial representation.

## Data Availability Statement

The raw data supporting the conclusions of this article will be made available by the authors, without undue reservation.

## Ethics Statement

The studies involving human participants were reviewed and approved by the French Ethical Committee for the research in sports science (IRB00012476-2021-05-02-82). The patients/participants provided their written informed consent to participate in this study.

## Author Contributions

BP-C, CB, and CM contributed to the conception and design of the study and performed the statistical analysis. CB performed the investigation and wrote the first draft of the manuscript. CS and PB organized the database, developed the experimental software, and the informatic programs for some data analyses (e.g., sigmoid analyses). BP-C, CB, CM, and VA contributed to the manuscript writing. All coauthors approved the submitted version.

## Conflict of Interest

The authors declare that the research was conducted in the absence of any commercial or financial relationships that could be construed as a potential conflict of interest.

## Publisher’s Note

All claims expressed in this article are solely those of the authors and do not necessarily represent those of their affiliated organizations, or those of the publisher, the editors and the reviewers. Any product that may be evaluated in this article, or claim that may be made by its manufacturer, is not guaranteed or endorsed by the publisher.
